# Executive function influences sedentary behavior: A longitudinal study

**DOI:** 10.15171/hpp.2016.29

**Published:** 2016-10-01

**Authors:** Paul D. Loprinzi, Allison Nooe

**Affiliations:** Physical Activity Epidemiology Laboratory, Department of Health, Exercise Science and Recreation Management, The University of Mississippi, University, MS 38677, USA

**Keywords:** Accelerometry, Cognition, Inhibition, Intention, Planning, Set-Shifting

## Abstract

**Background:** No study has evaluated the effects of executive function on follow-up sedentary behavior, which was this study’s purpose.

**Methods:** A longitudinal design was employed among 18 young adult college students (M_age_ = 23.7 years; 88.9% female). Accelerometer-determined sedentary behavior and physical activity, along with executive function, were assessed at baseline. Approximately 8 weeks later, re-assessment of accelerometer-determined sedentary behavior and physical activity occurred. Executive function was assessed using the Parametric Go/No-Go (PGNG) computer task. From this, 2 primary executive function outcome parameters were evaluated, including the Simple Rule and Repeating Rule.

**Results:** After adjusting for baseline sedentary behavior, age, gender, body mass index and baseline moderate-to-vigorous physical activity (MVPA), for every 25% increase in the number of correctly identified targets for the Repeating rule at the baseline assessment, participants engaged in 91.8 fewer minutes of sedentary behavior at the follow-up assessment (β = -91.8; 95% CI: -173.5, -10.0; P = 0.03). Results were unchanged when also adjusting for total baseline or follow-up physical activity.

**Conclusion:** Greater executive function is associated with less follow-up sedentary behavior.

## Introduction

Various theoretical models have been developed, implemented and evaluated to help guide physical activity promotion among all ages. Some of the commonly used theoretical models include the Transtheoretical Model, Health Belief Model, Social Cognitive Theory, and Theory of Planned Behavior (TPB). For a detailed review of these models, the reader is referred elsewhere.^1^

Here, we take a closer look at the TPB. The TPB postulates that behavioral intention is the most proximal predictor of behavioral engagement, with behavioral intention influenced by three antecedents, namely attitude, perceived social normal and perceived behavioral control. Previous research demonstrates utility for the TPB in explaining current physical activity,^2^ maintenance of physical activity,^3^ and sedentary behavior^4^ among adults. Like all theoretical models, however, the TPB is not without its shortcomings, which have been thoroughly discussed elsewhere.^5^ Among others, one potential limitation of the TPB is the reliance of a direct relationship between behavioral intention and behavioral engagement. There is some evidence to suggest that the relationship between behavioral intention and behavioral engagement may be moderated by several factors, such as gender.^2^ For example, the TPB constructs explained more variance in physical activity for women than men, and baseline intention demonstrated a stronger association with follow-up physical activity for women compared to men.^2^

Interestingly, there is also some emerging work demonstrating that an individual’s level of executive function may moderate the intention-behavior relationship.^6^ Executive function is often defined as “higher level” cognition that is used to describe subcomponents of cognitive function (e.g., attention). These subcomponents help to manage basic cognitive function to perform purposeful and goal-directed behaviors that are mediated by frontal lobe activity. Stated differently, individuals with optimal executive function have the ability to maintain an appropriate mental state to fulfill a future goal, which involves planning, filtering competing information, maintaining a goal despite distraction, and inhibiting goal-inconsistent responses.^7^ This suggests that a greater strength of association between intention and behavior may be observed among those with greater executive function.^6^ Not accounting for an individual’s level of executive function may be one reason for the relatively limited explained physical activity variance (~10%-20%) in the TPB.^2^ Of course, there likely are many other potential factors that may contribute to the relatively limited explained variance, such as the subjective assessment of physical activity in most of the TPB studies.

To our knowledge, only 1 study^6^ has examined the potential moderating role of executive function on the intention-behavior relationship and showed that intention was a stronger predictor of self-reported physical activity among young adults (N = 62) with greater executive function, as measured by the Go/No-Go test. The present study, written as brief study, is an extension of this study by evaluating whether executive function influences follow-up sedentary behavior among those with an intention to become more active in the future. Sedentary behavior is the outcome of interest in the present study given 1) the emerging research showing that sedentary behavior, independent of physical activity, is detrimentally associated with various health outcomes,^8-13^ and 2) that sedentary behavior has not been evaluated within the context of this topic (i.e., executive function and movement-related behaviors).

## Material and Methods

### Study design and participants

Consistent with our other related longitudinal work,^14^ 24 participants were recruited to participate in the present pilot longitudinal study. Among these 24, 6 did not return for the follow-up accelerometer assessment, resulting in an analyzed sample of 18 participants. As noted below, participants were eligible to participate if they had an intent to engage in more physical activity over the next 9 weeks and did not self-report engagement in regular, structured exercise at baseline (i.e., not currently active). All study procedures were approved by the authors’ institutional review board. Participant written consent was obtained prior to any data collection.

Assessments occurred at three time periods:

1) At baseline, participants completed a demographic questionnaire, the Go/No-Go computerized test to assess executive function (details below), self-reported assessment of physical activity (IPAQ-SF) and a questionnaire to assessed future intention to become more active. At this time, participants were also fitted with an ActiGraph GT9X accelerometer to wear for the subsequent 7 days.

2) One week after the baseline assessment, participants returned to the laboratory to hand in the accelerometer.

3) Eight weeks after returning the accelerometer, participants returned to the laboratory. At this time, they re-wore the accelerometer for another 7 consecutive days.

### Measurement of executive function

The Parametric Go/No-Go (PGNG) computer task was used to measure individual differences in executive function.^15^ This measure requires individuals to actively regulate responses to presented stimuli and either initiate responses quickly or inhibit their response. The executive function construct is multi-dimensional,^16^ and PGNG measures predominantly tap one facet of executive function that may be particularly pertinent to behavioral self-regulation: the ability to suspend prepotent responses to external cues. Functional imaging studies have documented associations between PGNG performance and activation in the prefrontal and anterior cingulate regions of the brain^17,18^; both structures have been implicated in behavioral self-regulation in humans.^19^ Detailed discussion of the factor structure and construct validity of the PGNG test is published elsewhere.^15,20^

As described elsewhere,^15^ there are three levels of the PGNG task, with the present study utilizing level 3, which is important for young healthy adult populations (consistent with the present sample) in order to remove potential ceiling effects.^15^ Utilizing computerized software, for level three, participants are presented with a series of 3 flashing letter targets (e.g., “r”, “s”, and “t”) intermixed with other letters (e.g., “a”, “c”, etc.), with each presented letter occurring at a rate of 500 ms. Within the 3 target level of PGNG, herein we evaluated 2 primary outcome parameters, including the Simple Rule and Repeating Rule. For the Simple Rule, participants are asked to press the space bar every time the target letter (e.g., “r”, “s”, or “t”) appears, with our evaluated outcome of this rule being the percent of correct target detection. For the Repeating Rule, participants are asked to press the space bar every time they see the target letter (e.g., “r”, “s”, or “t”), but only if the target letter is not repeating the previous target; our evaluated outcome for this rule was the percent of correct (non-repeating) target detection. For example, if the following letter sequence occurred, they would not press the space bar for the second “r” (a, t, r, p, d, r), but they would press the space bar twice (at “r” and “s”) during this sequence (a, t, r, p, d, s).

### Measurement of physical activity intention

At baseline, participants answered the following item^21^ using a paper-and-pencil survey; 1) I intend to be physically active on a regular basis (≥ 5 days/wk, 30 min/day of moderate-to-vigorous physical activity, MVPA) over the next 9 weeks. Notably, a “9 week” timeframe was used in this question because, as noted above, baseline accelerometry took 1-week to complete and then participants returned to the lab for re-assessment approximately 8-weeks later (8+1=9). Response options were: *strongly disagree*, *disagree*, *neither disagree nor agree*, *agree*, *strongly agree*. Only those answering “*strongly agree*” or “*agree*” were eligible to participate in this study. Notably, a sedentary behavior intention assessment was not evaluated given that such a psychometrically-robust assessment does not currently exist.

### Measurement of physical activity

*Self-Report.* Participants completed the short-form of the International Physical Activity Questionnaire (IPAQ). Specifically, they were asked, “During the last 7 days, on how many days did you do moderate or vigorous physical activity (response options: 0-7 days). On a typical one of these days, how much time in total did you usually spend engaging in moderate-to-vigorous physical activities?” This IPAQ Questionnaire has demonstrated evidence of reliability and validity in the adult population.^22^ Number of weekly minutes engaging in MVPA was calculated by multiplying the number of days by minutes of MVPA. Only participants who self-reported <75 min/wk of MVPA were eligible to participate in this study. Notably, at the time of this writing, there are no established guidelines for sedentary behavior for adults, and thus, we did not use baseline sedentary behavior as an exclusionary criteria.

*Accelerometry.* Physical activity was objectively measured using the ActiGraph GT9X accelerometer over a 7 day monitoring period, at both baseline and during the follow-up assessment (8 weeks after baseline). Participants were fitted with the accelerometer and worn on the mid-axillary line of the right hip at the level of the iliac crest. Accelerometers were initialized to a 1-minute epoch length. Sedentary behavior was defined as <100 counts/min.^23^ Light-intensity physical activity was defined as 100-1951 counts/min. MVPA was defined as ≥1952 counts/min.^24^ To monitor the amount of time the device was worn, nonwear was defined by a period of a minimum of 60 consecutive minutes of zero activity counts, with the allowance of 1-2 minutes of activity counts between 0 and 100.^25^ A valid day was considered as ≥10 h/day of wear time. Mean estimates of MVPA was determined by averaging the estimates from the valid days. Participants had to have at least 1 valid day of monitoring to be included in the analyses.^26^ Notably, all participants met this criteria.

### Statistical analyses 

Multivariable linear regression was used to examine the association between PGNG and sedentary behavior. As noted previously, the outcomes included the percent of correct target detection for the Simple Rule and Repeating Rule. For each of these outcomes, nested linear regression models were computed; Model 1: examining the association between PGNG and follow-up sedentary behavior (outcome variable); Model 2: same as Model 1 but controlling for baseline sedentary behavior; and Model 3: same as Model 2, but also controlling for age, gender, measured BMI and baseline MVPA. Statistical significance was established as *P *< 0.05. All analyses were computed in Stata (v. 12; College Station, TX).

## Results

Table 1 displays the characteristics of the analyzed sample. The mean age of the sample was 23.7 years and the majority were female (88.9%) and undergraduate students (61.1%). The mean follow-up period was 71 days.

Table 2 displays the regression analyses examining the association between baseline executive function (via PGNG) and follow-up sedentary behavior. Simple rule PGNG was not associated with follow-up sedentary behavior for any of the three evaluated regression models (unadjusted, minimally or extended adjust model); however, associations were in the expected (inverse) direction. Repeating rule PGNG were, however, statistically significantly inversely associated with follow-up sedentary behavior. For example, as shown in Model 3 for the Repeating rule, after adjusting for baseline sedentary behavior, age, gender, BMI and baseline MVPA, for every 1% increase in the number of correctly identified targets for the Repeating rule at the baseline assessment, participants engaged in 3.6 fewer minutes of sedentary behavior at the follow-up assessment (β = -3.6; 95% CI: -6.9, -.4; *P *= 0.03). Notably, results were similar when, instead of including baseline MVPA as a covariate, the model was adjusted for baseline counts per minute in the vertical axis (β = -3.5; 95% CI: -7.1, -.01; *P *= 0.04), light-intensity physical activity (β = -3.7; 95% CI: -6.9, -.4, *P *= 0.03), or total physical activity (light plus MVPA) (β = -3.5; 95% CI: -6.8, -.2, *P *= 0.03). Similarly, for Model 3 of the Repeating rule, results regarding the relationship between PGNG and follow-up sedentary behavior was similar when replacing the baseline MVPA covariate with the follow-up MVPA covariate (β = -3.7; 95% CI: -7.6, .07, *P *= 0.05) or follow-up total physical activity (β = -2.6; 95% CI: -5.1, -.07, *P *= 0.04). When expressed as a larger interval change in the fully adjusted model, for every 25% increase in the number of correctly identified targets for the Repeating rule at the baseline assessment, participants engaged in 91.8 fewer minutes of sedentary behavior at the follow-up assessment (β = -91.8; 95% CI: -173.5, -10.0; *P *= 0.03).

**Table 1:** Study variable characteristics of the analyzed sample (N = 18)

**Variable**	**Point estimate**	**SD**
Age, mean years	23.7	6.2
Female, %	88.9	
Education, %		
Undergraduate Student	61.1	
Graduate Student	38.9	
Race-Ethnicity, %		
White	77.8	
Black	16.7	
Other	5.6	
Body mass index, mean kg/m^2^	26.5	6.6
Sedentary behavior at baseline, mean min/day	591.6	169.7
Sedentary behavior at follow-up, mean min/day	534.7	96.4
Light-intensity activity at baseline, mean min/day	208.7	63.4
Light-intensity activity at follow-up, mean min/day	235.0	81.3
MVPA at baseline, min/day	29.2	18.0
MVPA at follow-up, min/day	23.9	18.1
Vigorous-intensity activity at baseline, mean min/day	0.89	1.6
Vigorous-intensity activity at follow-up, mean min/day	1.04	2.1
Counts per minute (vertical axis) at baseline, mean	259.4	107.2
Counts per minute (vertical axis) at follow-up, mean	258.5	93.5
Accelerometer wear time at baseline, mean min/day	829.6	149.5
Accelerometer wear time at follow-up, mean min/day	799.5	69.9
Valid days (10+ h/day) at baseline, mean	6.1	1.1
Valid days (10+ h/day) at follow-up, mean	4.6	1.5
3 Target Simple Rule, mean % correct	65.8	13.4
3 Target Repeating Rule, mean % correct	42.3	19.8
Follow-up period, mean days	71	13.9

Abbreviation: MVPA, moderate-to-vigorous physical activity.

**Table 2:** Regression analyses (β, 95% CI) examining the association between executive function (via PGNG) and follow-up sedentary behavior (outcome variable)

	**1% Increase in correct target detection**	
	**Simple rule**	**Repeating rule**
Model 1	-2.8 (-6.3, .65) [*P *= 0.10]	-2.3 (-4.5, -.06) [*P *= 0.04]
Model 2	-2.8 (-6.4, .7) [*P *= 0.11]	-2.6 (-5.1, .09) [*P *= 0.04]
Model 3	-4.1 (-8.8, .65) [*P *= 0.08]	-3.6 (-6.9, -.4) [*P *= 0.03]

Model 1, unadjusted; Model 2, adjusted for baseline sedentary behavior; Model 3, adjusted for baseline sedentary behavior, age, gender, body mass index and baseline MVPA. Interpretation of results (Model 3, Repeating Rule): After adjustments, for every 1% increase in the number of correctly identified targets for the Repeating rule at the baseline assessment, participants engaged in 3.6 fewer minutes of sedentary behavior at the follow-up assessment.

## Discussion

Research consistently demonstrates that physical activity is favorably associated with cognitive function,^27-30^ with mechanisms discussed elsewhere.^31^ Notably, recent evidence supports the association of sedentary behavior, independent of physical activity, on various health-related outcomes in adults, mostly cardiometabolic parameters,^9,10^ but also psychological^8^ and cognitive function^32^ outcomes as well. Interestingly, recent work^6^ suggests that, among other factors, an individual’s degree of executive function may be an important factor influencing whether behavioral intentions translate into behavioral initiation and maintenance. And previous work also demonstrates that the intention-behavior relationship is stronger for women compared to men, with the present study comprised of predominately women (88.9%). A potential greater strength of association between intention and behavior for women may be that women, compared to men, tend to utilize more behavioral (e.g., goal-setting, self-monitoring) and experiential (e.g., knowledge acquisition) processes of change.^33^ Within the context of the present study, a key component of executive function is behavioral self-regulatory ability, which is characteristic of behavioral processes of change. Taken together, future work would benefit by evaluating whether behavioral processes of change mediates the relationship between executive function and sedentary behavior.

The present study, to our knowledge, is the first to evaluate whether executive function influences sedentary behavior. The major finding of this pilot longitudinal study was that, indeed, young adults with greater executive function, as measured by the PGNG, had less follow-up sedentary behavior. This collective body of work suggests that the physical activity/sedentary behavior-cognitive function relationship is likely bi-directional. This potential bi-directional relationship has been highlighted in a recent review paper.^31^

Executive function is a central cognitive process, predominately influenced by frontal lobe activity, which may help to facilitate purposeful, goal-directed behavior and the inhibition of goal-inconsistent behaviors and responses. Further, executive function is often described as a process involving shifting between tasks or mental sets, updating and monitoring working memory, inhibition of prepotent responses, planning, and the coordination of multiple tasks.^16,34^ These descriptors provide plausibility for our observed association of executive function on follow-up sedentary behavior. That is, and as an example, individuals with greater executive function may have an increased cognitive flexibility to schedule, plan and execute activities that minimize prolonged sedentary behavior, while concurrently coordinating and managing multiple tasks (e.g., school, work) that may, via non-discretional time, inhibit inactivity and facilitate activity.

Limitations of this study include the small sample size and reliance on a singular cognitive task as a measure of executive function. Major strengths include the study novelty, longitudinal study design, and objective assessment of sedentary behavior. Future work, employing a larger, and more heterogeneous sample, is warranted.

In conclusion, the present pilot longitudinal study provides suggestive evidence that, among young adults, greater executive function is associated with less follow-up sedentary behavior. If confirmed by future research, then implementation of strategies to facilitate executive function may be an important strategy to minimize unfavorable changes sedentary behavior.

## Acknowledgments

No funding was used to prepare this manuscript.

## Ethical approval

This study was approved by the ethics committee at the University of Mississippi.

## Competing interests

We declare no conflicts of interest.

## Authors’ contributions

All authors were involved in the conceptualization of the study, revising the manuscript and interpreting the results. AN collected the data and PDL computed the analyses and drafted the first draft of the manuscript.

## References

[1] Buchan DS, Ollis S, Thomas NE, Baker JS (2012). Physical activity behaviour: an overview of current and emergent theoretical practices. J Obes.

[2] Plotnikoff RC, Lubans DR, Trinh L, Craig CL (2012). A 15-year longitudinal test of the theory of planned behaviour to predict physical activity in a randomized national sample of Canadian adults. Psychol Sport Exerc.

[3] Armitage CJ (2005). Can the theory of planned behavior predict the maintenance of physical activity?. Health Psychol.

[4] Prapavessis H, Gaston A, DeJesus S (2015). The Theory of Planned Behavior as a model for understanding sedentary behavior. Psychol Sport Exerc.

[5] Bagozzi RP (1992). The self-regulation of attitudes, intentions, and behavior. Soc Psychol Q.

[6] Hall PA, Fong GT, Epp LJ, Elias LJ (2008). Executive function moderates the intention-behavior link for physical activity and dietary behavior. Psychol Health.

[7] Loprinzi PD (2015). Physical activity is the best buy in medicine, but perhaps for less obvious reasons. Prev Med.

[8] Edwards MK, Loprinzi PD (2016). Effects of a sedentary behavior-inducing randomized controlled intervention on depression and mood profile in active young adults. Mayo Clin Proc.

[9] Loprinzi PD (2015). Sedentary behavior and predicted 10-yr risk for a first atherosclerotic cardiovascular disease (ASCVD) event using the pooled cohort risk equations among US adults. Int J Cardiol.

[10] Loprinzi PD (2015). Sedentary behavior and medical multimorbidity. Physiol Behav.

[11] Loprinzi PD (2015). Leisure-time screen-based sedentary behavior and leukocyte telomere length: implications for a new leisure-time screen-based sedentary behavior mechanism. Mayo Clin Proc.

[12] Loprinzi PD, Ford ES (2015). The association between objectively measured sedentary behavior and red blood cell distribution width in a national sample of US adults. Am J Epidemiol.

[13] Loprinzi PD, Pariser G, Ramulu PY (2014). Accelerometer-assessed sedentary and physical activity behavior and its association with vision among U.S. adults with diabetes. J Phys Act Health.

[14] Loprinzi PD, Sng E (2016). The association of changes in sedentary behavior on changes in depression symptomology: pilot study. J Behav Health.

[15] Langenecker SA, Zubieta JK, Young EA, Akil H, Nielson KA (2007). A task to manipulate attentional load, set-shifting, and inhibitory control: convergent validity and test-retest reliability of the Parametric Go/No-Go test. J Clin Exp Neuropsychol.

[16] Miyake A, Friedman NP, Emerson MJ, Witzki AH, Howerter A, Wager TD (2000). The unity and diversity of executive functions and their contributions to complex “Frontal Lobe” tasks: a latent variable analysis. Cogn Psychol.

[17] Hester R, Fassbender C, Garavan H (2004). Individual differences in error processing: a review and reanalysis of three event-related fMRI studies using the GO/NOGO task. Cereb Cortex.

[18] Watanabe M, Hikosaka K, Sakagami M, Shirakawa S (2002). Coding and monitoring of motivational context in the primate prefrontal cortex. J Neurosci.

[19] Heatherton TF (2011). Neuroscience of self and self-regulation. Annu Rev Psychol.

[20] Votruba KL, Langenecker SA (2013). Factor structure, construct validity, and age- and education-based normative data for the Parametric Go/No-Go Test. J Clin Exp Neuropsychol.

[21] Plotnikoff RC, Lubans DR, Costigan SA, Trinh L, Spence JC, Downs S (2011). A test of the theory of planned behavior to explain physical activity in a large population sample of adolescents from Alberta, Canada. J Adolesc Health.

[22] Craig CL, Marshall AL, Sjöström M, Bauman AE, Booth ML, Ainsworth BE (2003). International physical activity questionnaire: 12-country reliability and validity. Med Sci Sports Exerc.

[23] Matthews CE, Chen KY, Freedson PS, Buchowski MS, Beech BM, Pate RR (2008). Amount of time spent in sedentary behaviors in the United States, 2003-2004. Am J Epidemiol.

[24] Freedson PS, Melanson E, Sirard J (1998). Calibration of the Computer Science and Applications, Inc. accelerometer. Med Sci Sports Exerc.

[25] Troiano RP, Berrigan D, Dodd KW, Masse LC, Tilert T, McDowell M (2008). Physical activity in the United States measured by accelerometer. Med Sci Sports Exerc.

[26] Tudor-Locke C, Johnson WD, Katzmarzyk PT (2009). Accelerometer-determined steps per day in US adults. Med Sci Sports Exerc.

[27] Loprinzi PD (2016). Multimorbidity, cognitive function, and physical activity. Age (Dordr).

[28] Loprinzi PD (2016). Epidemiological investigation of muscle-strengthening activities and cognitive function among older adults. Chronic Illn.

[29] Loprinzi PD, Kane CJ (2015). Exercise and cognitive function: a randomized controlled trial examining acute exercise and free-living physical activity and sedentary effects. Mayo Clin Proc.

[30] Loprinzi PD, Herod SM, Walker JF, Cardinal BJ, Mahoney SE, Kane C (2015). Development of a conceptual model for smoking cessation: physical activity, neurocognition, and executive functioning. Res Q Exerc Sport.

[31] Loprinzi PD, Herod SM, Cardinal BJ, Noakes TD (2013). Physical activity and the brain: a review of this dynamic, bi-directional relationship. Brain Res.

[32] Falck RS, Davis JC, Liu-Ambrose T. What is the association between sedentary behaviour and cognitive function? A systematic review. Br J Sports Med. 2016. pii: bjsports-2015-095551. doi: 10.1136/bjsports-2015-095551. 27153869

[33] Fallon E, Hausenblas HA, Nigg CR (2005). The transtheoretical model and exercise adherence: examining construct associations in later stages of change. Psychol Sport Exerc.

[34] Friedman NP, Miyake A. Unity and diversity of executive functions: individual differences as a window on cognitive structure. Cortex. 2016. pii: S0010-9452(16)30107-1. doi: 10.1016/j.cortex.2016.04.023. PMC5104682 27251123

